# Spatial point patterns generation on remote sensing data using convolutional neural networks with further statistical analysis

**DOI:** 10.1038/s41598-022-18599-6

**Published:** 2022-08-22

**Authors:** Rostyslav Kosarevych, Oleksiy Lutsyk, Bohdan Rusyn, Olga Alokhina, Taras Maksymyuk, Juraj Gazda

**Affiliations:** 1 Department of Remote Sensing Information Technologies, Karpenko Physico-Mechanical Institute, NAS of Ukraine, Lviv, Ukraine; 2 Department of Telecommunications, Lviv Polytechnic National University, Lviv, Ukraine; 3 Technical University of Kosice, Kosice, Slovakia

**Keywords:** Ecosystem ecology, Forestry, Forestry, Computer science, Information technology, Scientific data

## Abstract

Continuous technological growth and the corresponding environmental implications are triggering the enhancement of advanced environmental monitoring solutions, such as remote sensing. In this paper, we propose a new method for the spatial point patterns generation by classifying remote sensing images using convolutional neural network. To increase the accuracy, the training samples are extended by the suggested data augmentation scheme based on the similarities of images within the same part of the landscape for a limited observation time. The image patches are classified in accordance with the labels of previously classified images of the manually prepared training and test samples. This approach has improved the accuracy of image classification by 7% compared to current best practices of data augmentation. A set of image patch centers of a particular class is considered as a random point configuration, while the class labels are used as marks for every point. A marked point pattern is regarded as a combination of several subpoint patterns with the same qualitative marks. We analyze the bivariate point pattern to identify the relationships between points of different types using the features of a marked random point pattern.

## Introduction

Nowadays, we observe a tremendous increase of the number of remote sensing devices and platforms such as low-orbit satellites, unmanned aerial vehicles, etc. With all these platforms we observe an exponential growth of the corresponding remote sensing data^1^. As result, so far we are observing a huge interest from the machine learning community to the analysis of remote sensing data, including object classification, pattern recognition, etc.^1–3^. Remote sensing (RS) image analysis allows us to solve a wide range of applied environmental tasks, such as monitoring of natural resources, landscapes, ecology, etc. It also provides opportunities for the analysis of the Earth’s surface in local and global scale, and make corresponding conclusions on ecosystems and their underlying biotic and abiotic controls^4–6^.

In addition to conventional 2D image cameras, there are also a number of alternative sensing solutions such as lidars or terrestrial laser scanning, which produce a scatter plot of points to reflect the footprint of ecological processes. These footprints can be detected through spatial point-pattern analysis (SPPA).

For the first time, the possibility of describing the ecological processes by the point patterns was indicated in^7,8^. Since that, research over the last two decades has undoubtedly proven that spatial point patterns and processes play an important role in understanding of plant and animal communities^9–13^. Being widely used in plant ecology, SPPA is frequently adopted to describe biotic interactions and interpret pattern process relationships^14^. Inhomogeneous summary statistics can quantify the impact of heterogeneity, while mark correlation functions can include trait and phylogenetic information in the analysis of multivariate spatial patterns. Furthermore, more refined point process models can be used to realistically characterize the structure of a wide range of patterns. The main objective of SPPA in ecology is to extract the information from corresponding patterns^15^. Identifying the type of interaction between the objects of environmental research will provide deeper understanding of the implicit processes that link them to build predictive dependencies of their behavior. To obtain the point pattern of investigated objects in some region a time-consuming investigation are necessary. Sometimes it is possible only with remotely received data. Therefore, it is important to develop new approaches for the analysis of remote sensing data, which allow to us to improve a process of identification of the existing dependencies among the elements of ecosystems.

The main contributions of this article are the following:

1. We propose a new method for the spatial point patterns generation by classifying remote sensing images using convolutional neural network.
2. We propose a new data augmentation scheme that uses a similarity between the images within the same part of the landscape for a limited observation time to improve the classification performance of convolutional neural network.
3. We analyze the bivariate point pattern to identify the relationships between different types of points using the features of a marked random point pattern.

The remainder of the paper is organized as follows. In “Background and related work” we present a background and related work on the environmental remote sensing and data analysis. In “Spatial point patterns generation by remote sensing data classification” we describe the details on our research workflow and main contributions of the paper. In “Simulation results and performance analysis” we present the experimental simulation results. In “Discussion and future research” we discuss the main outcomes and potential use cases for the proposed SPPA approach. Finally, we conclude the paper in “Conclusions”.

## Background and related work

### Recent trends in remote sensing

Environmental studies can be differentiated by two groups. The first one is the analysis of previously generated point patterns of the studied objects. The second is the qualitative selection of the desired objects in the image. The methods of the first group aim for a more accurate description of the environmental processes occurring in the target investigated area, while the methods of the second group aim for more accurate detection of objects. Both groups require a labor-intensive data collection to obtain corresponding spatial point patterns and accurately determine the target features of each object. Thus, comprehensive approaches for environmental monitoring are getting momentum so far^16–19^. In^16^, authors describe main reasons why RS has become an important source of data, and present the different types of sensors and platforms that have been used to display the diversity of forest variables.

The rapid development of RS technologies supports forest management and conservation in those parts of the world where environmental issues are most critical. For example, in^17^ a methodology for continuous monitoring and estimation of land cover and land change areas have been tested. This methodology is based on the recent advancements in the field of environmental RS, using algorithms for time series analysis and estimation protocols. The research^18^ is devoted to modelling and predicting the species richness. By diversifying multiple sensors, which examine relationships over different temporal and spatial scales, authors have focused on the normalized difference vegetation index (NDVI) from passive sensors. NDVI has been associated with net primary productivity and has been hypothesized to quantify species richness and diversity based on the species energy theory^19–26^. In particular, in^19^ authors review techniques that are currently used for monitoring, including close-range RS, airborne and satellite-based approaches. The implementation of optical, RADAR and LiDAR RS-techniques to assess spectral traits/spectral trait variations (ST/STV) is described in detail. They found that ST/STV can be used to record indicators of ecosystem state based on RS. Therefore, the ST/STV approach provides a framework to develop a standardized monitoring concept for ecosystem condition indicators using RS techniques that is applicable to future monitoring programs. In addition, in^19^ authors analyze two different approaches: empirical modelling and physical modelling for the estimation of ST and trait variations of ecosystem condition from RS data. Authors have investigated that empirical models based on statistical relationship between one or more biotic traits and one or more spectral variables that are widely used for the quantitative modelling of biophysical vegetation parameters on a global scale.

Analyzing the above-mentioned related research, we can conclude that currently there is a lack of approaches that would directly link the formation and modeling of biotic processes in ecosystem based on random point patterns generated by remote sensing data. It is worth noting that both spatial point patterns and remote sensing images are able to work in different scales. It is achieved by the identification of events and territories, as well as by the different image resolutions. Therefore, we use this similarity in our methodology of environmental research.

### Classification of remote sensing data

Nowadays, it is relatively easy to obtain an enormous amount of remote sensing data from open access or commercial databases^27,28^. However, the analysis of remote sensing images is time consuming and may depend on human subjective assessment. Recently, various automated techniques of remote sensing image analysis have been used to facilitate these tasks^29–39^. Both classical methods of image processing^29–35^ and more modern methods based on convolutional neural networks (CNNs)^36–39^ provide a satisfactory result. Nevertheless, the CNN based algorithms often achieve higher performance in most image analysis tasks. In particular, most remote sensing tasks involve selection of quantitative characteristics of the studied objects^32,33^, which includes image segmentation and classification. CNN also offers very fast processing in the inference stage, assuming that the model is well trained initially. Nevertheless, the training is needed also for other known classification approaches, including support vector machine, random forest, k-nearest neighbors, etc.^34,35^.

Despite the progress in classification techniques, the training phase and the dataset quality are still key factors in achieving high classification accuracy of CNN. Its qualitative construction requires considerable effort, especially for remote sensing tasks. First, due to the specifics of their generation, most of images are distorted by the atmosphere. Another factor that has a significant impact is the image resolution. Such aspects, significantly limit the scope of problems that can be considered and the choice of methods for solving them. High-resolution images (∼ 1 m per pixel) of the desired object are required for local scale tasks, while lower-resolution images are sufficient for solving global scale tasks. In both cases, such images are quite large, and result in considerable computational complexity of their processing. High-quality high-resolution images are rarely available, so in most cases, images with a resolution of approximately 10 m per pixel are used for monitoring tasks. This aspect significantly limits the ability to classify required objects that might be represented by only a few pixels and makes it difficult to prepare training samples of different image classes due to their similarity. It is well known, that the accuracy of qualitative classification of images is directly proportional to the size of the training dataset. The well-known ImageNet^35^ image dataset, which is frequently used as a benchmark for modern classification tools, contains more than one million labeled images.

Summarizing all abovementioned issues, we can conclude that CNN allows to perform initial training on known dataset or use the previously trained weights (transfer learning) with further fine-tuning on the target data. A significant limitation is that features of the target images must match the imported features, which is not always the case. Therefore, the generation of CNN training data for the classification task and their further analysis remain an unsolved urgent problem in environmental studies.

### Spatial point patterns analysis in environmental studies

Tasks that require a statistical description of the sequence of events that occur at certain points in space often arise in many fields of technology and economics. Additionally, knowledge of the characteristics of the spatial distribution patterns of natural resources is crucial for developing an understanding in many fields of ecology. In the simplest one-dimensional case, the sequence of random events occurring in time can be characterized by random moments of their appearance. Such a sequence of events is often called a random flow^36^ or a random point process (RPP). For a two-dimensional case, the presence of related events is even more important, provided that these events occurred within a short period of time. The analysis of RPP can establish arrangement patterns of random events and hence reveal their influence on each other and its type: mutual or asymmetric, or none of these. Establishing the properties of the process allows us to gain more information about the object itself.

**Figure 1:**
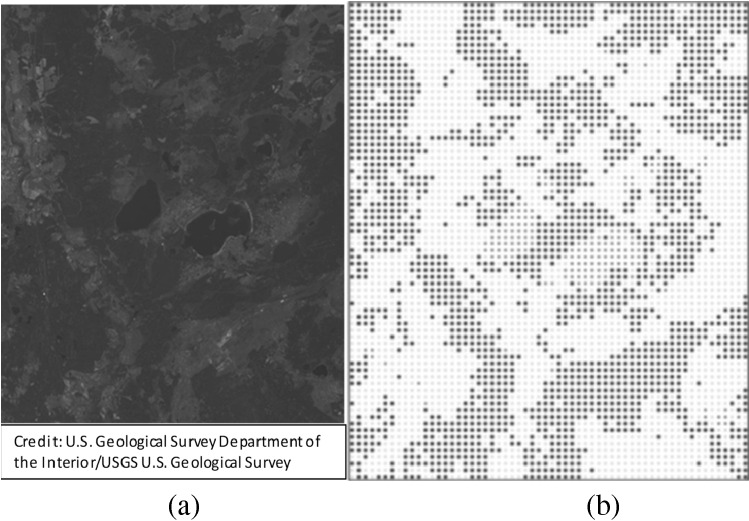
Remote sensing image (**a**)^28^, and its point-pattern (**b**).

Historically, object analysis using RPP was first applied in disciplines where the object could have been located on the map: astronomy, seismology, economics, sociology, materials science, etc.^37,38^. Therefore, over the last two decades, SPPA has become increasingly popular in ecological and other research^39^. The division of the input image into points and their subsequent classification allows the generation of various points, which correspond to different classes. By substituting each image fragment with a point at its center, we can produce a configuration displaying the location of fragments of the same class, as shown in Fig. 1^28^.

Recently, ecologists have increasingly used such datasets to quantify the characteristics of observed spatial patterns. Most of the research aimed at deriving the hypotheses on underlying processes or testing hypotheses derived from ecological processes or theory. One of the main characteristics of the observed point configuration is the type of point location. The cluster, regular or random configuration types are distinguished according to the nature of the interaction between them. For cluster point configuration, the presence of interaction is assumed, causing the attraction of elements to each other, while for regular, the repulsion from elements to each other. Finally, the random configuration assumes no interaction between its elements. The homogeneous Poisson point process is a fundamental model for RPP, and it also serves as a basis for more complex models. This process reflects the concept of "absolute" randomness, which is expressed by the absence of a definite internal structure of the set of points that correspond to this point pattern. A number of tests have been developed to test the hypothesis of complete spatial randomness. The most commonly used is the Clark-Evans test^40^, which is based on examining the normal distribution of the standardized average distance to the nearest neighbor. Another way to analyze RPP is to calculate its second-order characteristics, including the K-function, g-function, J-function and others^36,41,42^.

Wider opportunities are provided in the case of the simultaneous analysis of a mutual arrangement of two or more species. Examining biological objects, for instance, makes it possible to establish interspecific relations. Conventionally, point configurations are formed by manually plotting the locations of events or objects of study. This is a time-consuming process requiring knowledge of the exact distances between the objects of study, which are often distributed over large areas. Therefore, aerial photography images are the preferable source for the generation of spatial point patterns. Although they are detailed enough to reflect the location of the studied objects, they are suitable only for the analysis of local areas and relevant processes. On the other hand, in our opinion, images obtained by satellites are well suited for the study of more global processes. Therefore, in this paper, we propose a framework that uses satellite remote sensing data transformed to point patterns with the aim of describing biotic interactions on a global scale.

## Spatial point patterns generation by remote sensing data classification

### Remote sensing data augmentation based on the spatiotemporal similarity between images

Currently, the CNN based models are considered as the most effective in image classification, which outperform other methods in training, inference accuracy and generalization^43–46^. To achieve a minimal training error CNN parameter must well align with training data. In the vast majority of practical tasks, the size of the training sample is insufficient, so that it is necessary to increase it. The traditional way to increase the training and test sets of images is the data augmentation^47–49^.

Typically, the methods of data augmentation apply the various transformations to image data: rotation, shift, scaling, changing the color palette and their combinations^47–49^. In general, the use of data augmentation can increase the accuracy of classification by 5–10%. However, existing data augmentation solutions the gain in accuracy is observed only in case of the significant difference between images. This is not applicable for remote sensing image fragments due to their proximity. In this case, augmentation produces copies of image fragments of the original sample and does not affect the classification accuracy (Table 1).

**Table 1:** Comparison of classification accuracy with and without data augmentation.

Training sample size	Accuracy, %, augmentation/no augmentation	Training time, s, augmentation/no augmentation
11,792	80/83	30/105

To increase the training sample size, we take advantage of the unique property of remote sensing images, namely, the periodicity of receiving such images. It is especially applicable for satellite imagery but can also generally be implemented for aerial imagery. Having the period of rotation of satellites around the Earth and the period of rotation of the Earth, we can collect a series of images of the target area of interest during a specified period of time. These will be similar images, each differently affected due to circumstances of remote sensing, and primarily by meteorological factors. Accurate distinguishing of the same area of the surface in these images is an effective kind of transformation that must be applied to restore the original image. The particular nature of the transformation does not matter, but the fact that images are different is essential. By selecting one of the images as the source and marking its fragments according to the set of preset classes, we can assign the same classes to the corresponding fragments of other images. In this way, an extended set of marked fragments is produced that can be used for both the training and test sets.

### Dataset preparation

Training and test image samples were taken from the images of the Shatsk National Nature Park (SNNP), which were obtained by the Landsat 8 satellite in the visible spectral range^28^. The data set was collected during 2017–2020. Images obtained during June–September were selected for the research, as they fully depict the vegetation of the investigated region. The images display a section of SNNP with an area of 450 km^2^. The size of the original image is 3420 × 4380 pixels with a precision of approximately 10 pixels per meter. Based on the selected image, training, test and validation samples were generated with image patches of 10 × 10 pixels. The number of fragments in each category is 11,792, 1664, and 482, respectively. To extend these samples, it was proposed to supplement them with corresponding fragments present in a number of other images, since the generated dataset reflects the same area of the Earth’s surface. For that purpose, we assigned class labels to such additional fragments according to the initial training sample and then conducted CNN training. As result, we have extended a dataset by classifying the fragments of supplementary images to the 47,213, 10,443 and 2182 fragments, respectively. Overall, by supplementing a number of images, we have further produced a following data samples: training—186,027; test—45,019; validation—22,349.

### CNN training for the classification of remote sensing images

To classify the remote sensing images, we have chosen a powerful CNN configuration (EffNet)^50^ and the simple CNN configuration (LeNet)^51^. The training has been conducted by the following parameters: optimizer—Adam; batch size—256; number of epochs—30; number of classes—6; learning rate—not defined explicitly. As shown in Table 2, an unexpected similarity has been observed in the classification results of different CNNs. Specifically, for the above-mentioned EffNet and LeNet, the corresponding results differ by no more than 2%. This can be explained by the domination of large-scale features in the investigated images. Therefore, for the current remote sensing images, the more complex CNN architectures, which are available nowadays, will not provide a sufficient advantage, especially considering the inevitable increase of computational complexity. Moreover, regardless of the CNN architecture, the proposed approach of data augmentation has improved the accuracy of classification by more than 10% (Table 2).

**Table 2:** Classification accuracy for different CNN architectures.

Data set capacity	Accuracy (top-1) %		Accuracy (top-5) %	
	EffNet	LeNet	EffNet	LeNet
11,792/1664/482	96/80/68	80/76/65	100/98/79	100/99/75
47,213/10,443/2182	96/86/77	86/88/67	100/99/90	99/99/89
186,027/45,019/22,349	97/90/89.7	87/89/88.9	100/99/95	99/99/93

## Simulation results and performance analysis

**Figure 2:**
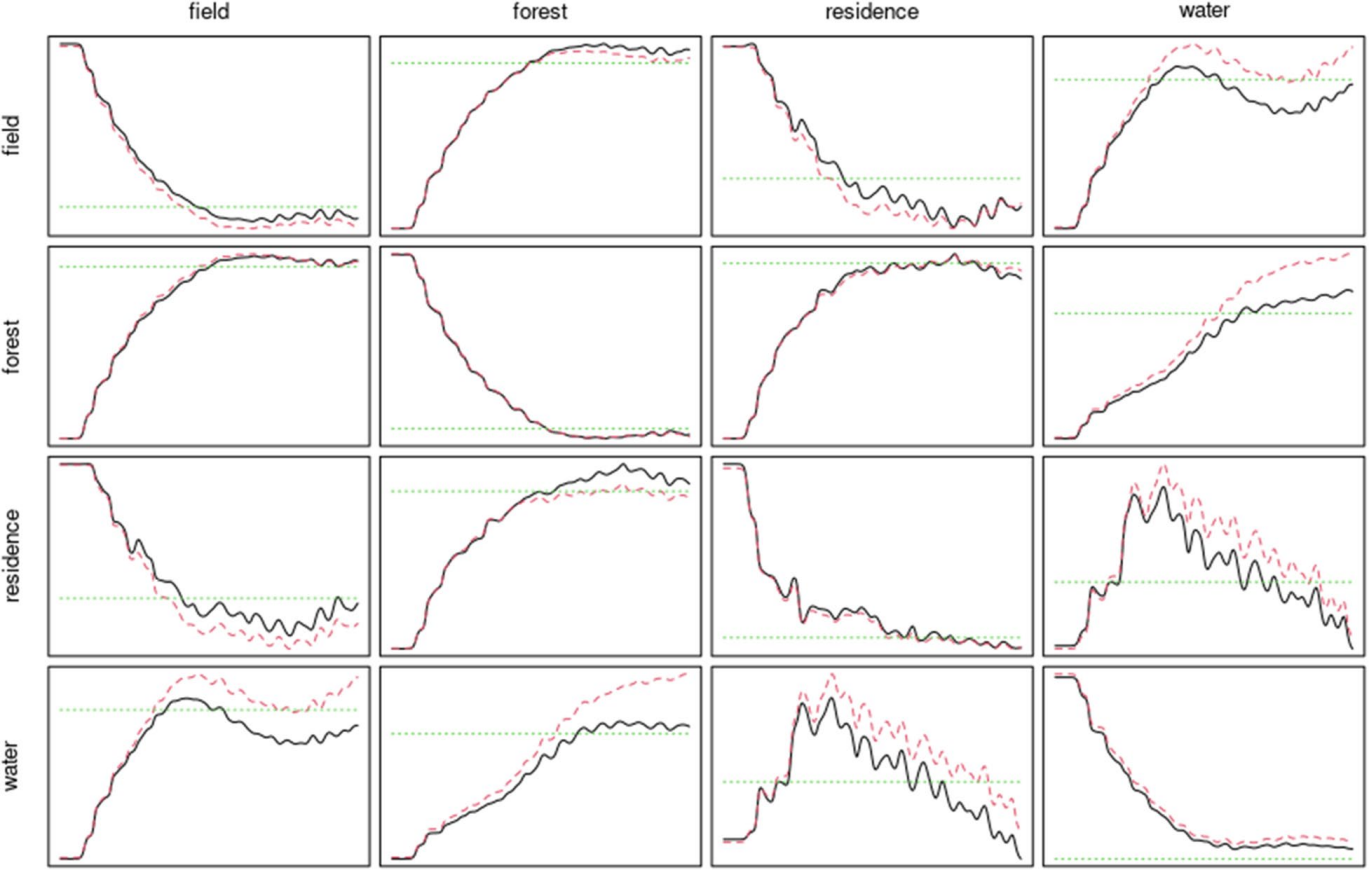
An array of mark connection functions for point patterns from Fig. 1b.

To analyze several configurations of points, the properties and characteristics of marked point processes are considered, which differ from the ordinary ones by the inclusion of an additional parameter—a marker that describes both qualitative and quantitative characteristic. To study these properties, we use the two-dimensional analogy of ordinary point processes characteristics, such as *K, g, J*—functions and others^36,42^. Let’s define the corresponding expressions for these functions. The expression for the *K*-function in the case of a single point configuration is given by:

$$\lambda K(r) = E_{o} (N(b(o,r)/\{ o\} ),$$

where *λ* is the number of points per unit area, and *K*(*r*) denotes the average number of points in a circle with radius *r* whose center is located at a typical point that is excluded from consideration. In the case of multiple point configurations, this function looks similar:

$$\lambda_{i} K_{ij} (r) = E_{oi} (N_{j} (b(o,r)/\{ o\} ),$$

where *λ*_*i*_ is the number of points of type *i* per unit area, and *K*_*ij*_(*r*) is the average number of points of type *j* in a circle of radius *r*, the center of which is located at a typical point of type *i*. When considering all pairs of points separated by distance *r*, mark connection functions *p*_*lm*_(*r*) yield the conditional probability of the first point being of type *l* and the second of type *m*^10,41^. It gives a measure of dependency between two such points of the process a distance *r* apart. If the marks attached to the points are independent and equally distributed, then *p*_*lm*_(*r*) = *p*_*l*_ pm, where *p*_*l*_ denotes the probability of the point being of type *l*. Otherwise, *p*_*lm*_*(r)* > *p*_*l*_ pm suggests a positive association between the two types, while *p*_*lm*_*(r)* < *p*_*l*_ pm indicates a negative association. The Mark correlation function *k*_*mm*_*(r)* is a second-order summary statistic adapted to quantitatively marked patterns. *k*_*mm*_*(r)* visits all pairs of points separated by distance *r*, estimates the mean of the product of their marks, and divides this by the corresponding quantity taken over all pairs of points. It can be used to detect correlations in the case of mutual stimulation or inhibition of individuals^42^. If there is no interaction between points, then the function is equal to 1 for all distances. If *k*_*mm*_*(r)* > 1, points located at distance *r* indicate a mutual stimulation or positive correlation. An opposite situation, *k*_*mm*_*(r)* < 1, indicates mutual inhibition between points^42^. An example of applying the proposed approach to the analysis of remote sensing images is shown in Fig. 2. It displays the relationships between the components of a random point pattern that contains 4 constituents. These dependencies were built based on the assumption of the mutual presence of elements of one component in the vicinity of a typical point of another component. The number of elements of one type in the vicinity of another can serve as a measure of the compatibility of these types. In particular, the dependencies between point configurations for pairs of classes were constructed. Such dependencies may show, for example, trends in the development of the region’s vegetation, expansion or contraction of areas of habitat of individual species.

The study of labeled point configurations using the characteristics of the 2nd order allows us to draw certain conclusions about a particular species as well as interspecific connections. Obviously, we deal with traditional characteristics when performing the analysis of point configuration of one/single type; hence, we can focus exclusively on labeled point configurations. A number of point configurations were selected for the SNPP study area. Analysis of these configurations can explain the dynamics of the ecosystem of this region. Any significant works that might affect the environment are prohibited in the territory of SNPP; hence, the formed point images reflect the natural processes that take place there. This is especially true for afforestation. A number of conclusions can be drawn from a series of graphs showing the relationship between forests and other objects, as presented in Fig. 2.

**Figure 3:**
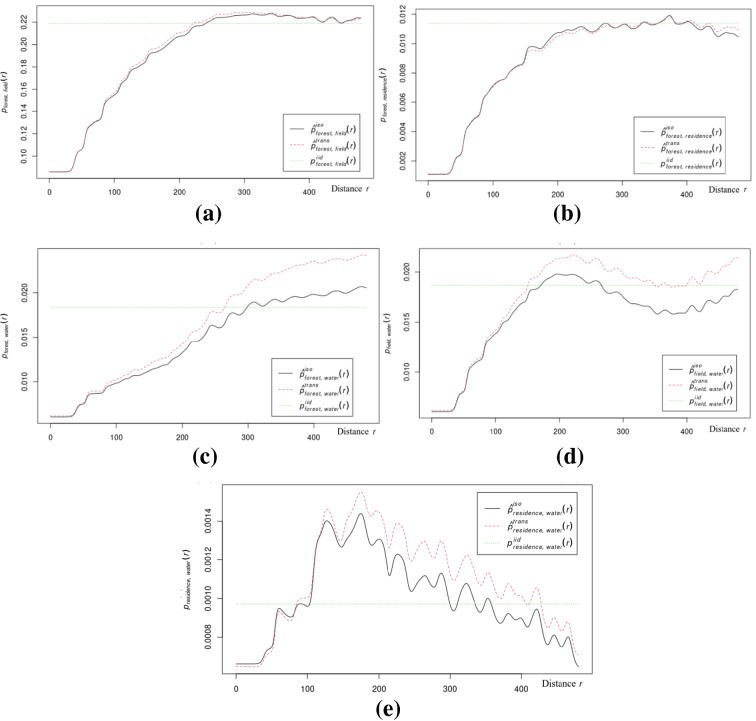
Plots of mark connected functions for bivariate marked point pattern. (**a**) “forest-water”; (**b**) “forest-residence”; (**c**) “water-forest”; (**d**) “water-field”; (**e**) “water-residence”.

Let us consider these images individually (Fig. 3). The first image (Fig. 3a) shows the relationship between marked configurations describing areas of forest and fields. The level of dependency is the probability of the presence of elements of both at a certain distance. Based on the plot, we can conclude that even at distances up to 2 km (mark 200 on the graph), the probability of their joint occurrence is quite low (0.2). Another plot (Fig. 3b) indicates a low (0.01) probability of finding buildings in the forest, which is as expected for restricted areas. Similarly, comparing dependency plots of water-field and water-forest pairs (Fig. 3c, d), we can conclude that water resources are mostly surrounded by forests. The last plot (Fig. 3e) indicates the probability of finding buildings near the water.

## Discussion and future research

Statistical analysis of remote sensing data for a particular region is both time consuming and resource-intensive task. Usually it requires to estimate the volumes of available species and their key characteristics and then evaluate their growth reliability. Today, it is possible to collect relevant data with the help of advanced technologies of remote sensing, satellite and aerial imaging. Nowadays, remote sensing of the Earth surface has multidisciplinary practical value, where the ecology of the landscape and associated statistical methods are becoming increasingly important^41^.

Among various available approaches for remote sensing data analysis the CNN-based solutions are the most promising ones in terms of accuracy. A significant part of research on CNN and other deep learning models is devoted to the problem of acquisition of the training, test and validation data. Therefore, the proposed approach for spatial point patterns generation opens a new perspective area of investigation that can significantly increase the potential of the remote sensing image analysis. The use of remote sensing data allows to analyze problems of different scales, but in most cases, it comes down to extracting the target information from the image. Interpretation of such information is a simple statistical analysis, such as calculating the number, area or perimeter, and other parameters. This does not reveal the full potential of such data. For the purpose of deeper analysis, the transformation of such data into spatial point images is proposed. Their apparatus can establish both simple quantitative characteristics such as intensity, and more complex, which reflect the relationships between different parts of the data. The results of our research give an example of establishing such relationships between different classes of objects on the example of SHNP.

Typically, the point patterns are formed based on the exact location of each element in the image. However, in our case, we artificially create an element of the point image as the center of the image fragment. Proposed approach is different from the fully stochastic solutions, which are known in the literature, such as^52^ where the point images are generated based of forest fires and the element of the point image is chosen as the center of the fire area. Among other examples: the original dataset is a pattern of small blobs, and the points are the blob centres; the original dataset is a collection of line segments, and the points are the endpoints, crossing points, midpoints etc.; the original dataset is a space-filling tessellation of biological cells, and the points are the centres of the cells. All cases prove that point process methodology can be more powerful or more flexible than the existing methodology for the raw image data. Nevertheless, the origin of the point pattern may lead to artefacts which must be considered in the analysis^53^. In our research, we have shown a possibility of the application of spatial point patterns generation for the analysis of real data based on the example of SNNP satellite images.

In future research, there are still open areas for SPPA general adoption. In particular, this is due to some skepticism in using static point images to describe and analyze a dynamic ecosystem. Moreover, there is still an incomplete understanding of the correspondence between random processes that simulate the location of random point configurations and underlying ecological processes in the study region. Despite the obvious successes of both the theory of point processes and its numerous applications, including environmental monitoring, there are a number of problems limiting its wider application. First, apart from their complexity, there is only a small number of ready-to-use zero-models of RPPs to hand, as well as tests for their verification on real data. This means that each application requires its own mathematical apparatus for formalization.

Nevertheless, the advantage of using SPPA for the real data analysis is not only its ability to describe existing objects but also the ability to reflect existing connections between them. The latter, as already mentioned, is the main task of environmental monitoring. While the traditional and widely used SPPA apparatus for the analysis of real objects accounts only for their location, it is also important to account for their individual characteristics by applying models of marked random processes and determining their parameters.

Finally, there is still a wide area of research related to the usage of advanced CNN and other deep learning applications in combination with spatial point patterns, which may result in significant boost of the remote sensing data analysis.

## Conclusions

The tasks of environmental monitoring require improvements to existing methods as well as the development of new methods for the analysis of remote sensing data. The most common source of such information is remote sensing images of the Earth’s surface. The essential step in the analysis of such images is their classification. Most effectively, at present, this problem is solved by using convolutional neural networks. However, for such an approach to be successful, large training and test data sets are needed. To enhance such data, it is proposed to supplement them with additional augmented data, which is similar to the original data. Proposed data augmentation scheme outperforms the current best practices by at least 7% in classification accuracy. In addition, the methodology for the remote sensing images analysis is proposed based on a combination of image fragment classification by CNN and determining the relationship between the components of the marked random point field generated based on these fragments. The proposed approach allows to reflect the relationships between structural elements of the investigated areas of the ecosystem.

## Data Availability

The data presented in this study are available on request from the corresponding author. The data are not publicly available because they are a fragment of other publicly available data.
